# Supplementary material for: Hot spot prediction in protein-protein interactions by an ensemble system
Source: BMC Syst Biol. 2018 Dec 31;12(Suppl 9):132. doi: 10.1186/s12918-018-0665-8 (PMC6311905; doi:10.1186/s12918-018-0665-8)
Supplement: Supplementary file 2 — All relevant MATLAB code. A simple Matlab implement of our predictor. (ZIP 19,742 kb) [file 12918_2018_665_MOESM2_ESM.zip › Additional file 2/Read ASA/ASA website/dbMPIKT/4P23C.html]

 NetSurfP - Protein Surface Accessibility and Secondary Structure Predictions

  

|  |  |  |
| --- | --- | --- |
|  |  | NetSurfP - Protein Surface Accessibility and Secondary Structure Predictions  Technical University of Denmark |

  


---

```
# For publication of results, please cite:
# A generic method for assignment of reliability scores applied to solvent accessibility predictions.
# Bent Petersen, Thomas Nordahl Petersen, Pernille Andersen, Morten Nielsen and Claus Lundegaard
# BMC Structural Biology 2009, 9:51 doi:10.1186/1472-6807-9-51
#
# Column 1: Class assignment - B for buried or E for Exposed - Threshold: 25% exposure, but not based on RSA
# Column 2: Amino acid
# Column 3: Sequence name
# Column 4: Amino acid number
# Column 5: Relative Surface Accessibility - RSA
# Column 6: Absolute Surface Accessibility
# Column 7: Z-fit score for RSA prediction
# Column 8: Probability for Alpha-Helix
# Column 9: Probability for Beta-strand
# Column 10: Probability for Coil
E I  4P23C                  1    0.607 112.277  -0.765   0.003   0.003   0.994
E E  4P23C                  2    0.622 108.733   0.694   0.004   0.085   0.910
E A  4P23C                  3    0.407  44.818  -0.274   0.020   0.205   0.775
E D  4P23C                  4    0.661  95.265   0.089   0.019   0.141   0.840
B H  4P23C                  5    0.262  47.585   0.027   0.021   0.279   0.699
E V  4P23C                  6    0.295  45.403   0.192   0.003   0.718   0.279
B G  4P23C                  7    0.153  12.065   0.532   0.001   0.900   0.099
B T  4P23C                  8    0.138  19.210   0.659   0.001   0.900   0.099
B Y  4P23C                  9    0.070  14.980   0.504   0.001   0.900   0.099
B G  4P23C                 10    0.043   3.360  -0.486   0.002   0.816   0.182
B I  4P23C                 11    0.027   5.051   0.721   0.001   0.900   0.099
B S  4P23C                 12    0.092  10.818  -0.280   0.001   0.959   0.040
B V  4P23C                 13    0.042   6.409   0.696   0.001   0.900   0.099
B Y  4P23C                 14    0.164  35.090   0.277   0.003   0.718   0.279
E Q  4P23C                 15    0.501  89.532  -0.906   0.004   0.420   0.576
E S  4P23C                 16    0.625  73.227  -2.592   0.005   0.045   0.951
E P  4P23C                 17    0.597  84.714  -2.835   0.005   0.015   0.979
E G  4P23C                 18    0.412  32.401  -0.937   0.005   0.045   0.951
B D  4P23C                 19    0.280  40.362  -1.016   0.004   0.138   0.858
E I  4P23C                 20    0.478  88.356  -0.059   0.005   0.262   0.733
B G  4P23C                 21    0.119   9.397  -0.268   0.004   0.616   0.381
B Q  4P23C                 22    0.101  18.128   0.051   0.001   0.959   0.040
B Y  4P23C                 23    0.166  35.453   0.094   0.001   0.959   0.040
B T  4P23C                 24    0.153  21.152   1.434   0.000   0.983   0.017
B F  4P23C                 25    0.065  13.045   0.725   0.001   0.959   0.040
B E  4P23C                 26    0.060  10.447  -0.208   0.001   0.959   0.040
B F  4P23C                 27    0.076  15.313   0.655   0.001   0.900   0.099
E D  4P23C                 28    0.302  43.561   0.662   0.005   0.262   0.733
E G  4P23C                 29    0.344  27.089  -0.910   0.018   0.019   0.964
E D  4P23C                 30    0.493  71.070   0.142   0.020   0.205   0.775
B E  4P23C                 31    0.196  34.154   0.227   0.022   0.552   0.426
B L  4P23C                 32    0.105  19.299   0.916   0.018   0.846   0.136
B F  4P23C                 33    0.062  12.484   0.842   0.011   0.918   0.071
B Y  4P23C                 34    0.133  28.358   0.783   0.011   0.918   0.071
B V  4P23C                 35    0.043   6.594   0.559   0.011   0.918   0.071
B D  4P23C                 36    0.168  24.223  -0.389   0.018   0.846   0.136
B L  4P23C                 37    0.187  34.185  -0.121   0.022   0.359   0.619
E D  4P23C                 38    0.576  82.958   0.983   0.019   0.141   0.840
E K  4P23C                 39    0.555 114.246   0.432   0.018   0.047   0.935
E K  4P23C                 40    0.488 100.443  -0.823   0.018   0.088   0.893
E E  4P23C                 41    0.393  68.570   0.191   0.021   0.451   0.528
B T  4P23C                 42    0.171  23.690  -0.083   0.023   0.655   0.322
B V  4P23C                 43    0.213  32.738   0.122   0.021   0.756   0.223
B W  4P23C                 44    0.139  33.357  -0.001   0.021   0.756   0.223
B M  4P23C                 45    0.230  46.103  -0.273   0.022   0.552   0.426
B L  4P23C                 46    0.130  23.766  -0.488   0.064   0.216   0.721
E P  4P23C                 47    0.376  53.312  -0.730   0.268   0.043   0.689
E E  4P23C                 48    0.418  73.077  -0.715   0.455   0.046   0.498
B F  4P23C                 49    0.282  56.557  -0.549   0.522   0.016   0.462
B G  4P23C                 50    0.160  12.608  -1.261   0.522   0.016   0.462
E Q  4P23C                 51    0.504  89.943   0.027   0.522   0.016   0.462
E L  4P23C                 52    0.297  54.417  -0.058   0.622   0.015   0.363
B A  4P23C                 53    0.135  14.844  -0.173   0.622   0.015   0.363
E S  4P23C                 54    0.352  41.208  -0.685   0.561   0.047   0.393
E F  4P23C                 55    0.362  72.673  -0.057   0.605   0.105   0.290
E D  4P23C                 56    0.317  45.723  -0.029   0.502   0.102   0.396
B P  4P23C                 57    0.282  40.002  -0.027   0.701   0.107   0.192
E Q  4P23C                 58    0.393  70.172  -0.987   0.605   0.105   0.290
E G  4P23C                 59    0.375  29.520  -0.757   0.561   0.047   0.393
E G  4P23C                 60    0.392  30.843  -1.207   0.354   0.048   0.598
E L  4P23C                 61    0.402  73.679  -0.966   0.278   0.093   0.628
E Q  4P23C                 62    0.480  85.799  -0.933   0.118   0.150   0.732
B N  4P23C                 63    0.282  41.358  -1.010   0.069   0.386   0.545
B I  4P23C                 64    0.295  54.630  -0.158   0.021   0.451   0.528
B A  4P23C                 65    0.227  24.993  -0.982   0.023   0.655   0.322
B V  4P23C                 66    0.223  34.321  -0.067   0.021   0.756   0.223
B V  4P23C                 67    0.058   8.884   0.422   0.021   0.756   0.223
E K  4P23C                 68    0.331  68.046   0.338   0.021   0.756   0.223
B H  4P23C                 69    0.263  47.840  -0.961   0.021   0.756   0.223
B N  4P23C                 70    0.272  39.806  -0.194   0.079   0.592   0.329
B L  4P23C                 71    0.252  46.178  -0.506   0.074   0.484   0.442
E G  4P23C                 72    0.394  31.016  -1.005   0.074   0.484   0.442
E V  4P23C                 73    0.322  49.461  -1.013   0.148   0.418   0.435
E L  4P23C                 74    0.335  61.302  -1.219   0.135   0.317   0.548
E T  4P23C                 75    0.350  48.490  -0.798   0.125   0.227   0.648
E K  4P23C                 76    0.407  83.699  -1.399   0.118   0.150   0.732
E R  4P23C                 77    0.356  81.638  -1.034   0.199   0.152   0.649
E S  4P23C                 78    0.319  37.434  -1.109   0.191   0.086   0.723
E N  4P23C                 79    0.496  72.570  -1.249   0.268   0.043   0.689
E S  4P23C                 80    0.485  56.901  -0.929   0.268   0.043   0.689
E T  4P23C                 81    0.390  54.051  -1.474   0.191   0.086   0.723
E P  4P23C                 82    0.410  58.236  -1.199   0.118   0.150   0.732
E A  4P23C                 83    0.535  59.012  -1.544   0.125   0.227   0.648
E T  4P23C                 84    0.450  62.443  -1.124   0.064   0.216   0.721
E N  4P23C                 85    0.433  63.333  -1.265   0.064   0.216   0.721
E E  4P23C                 86    0.469  81.987  -1.568   0.019   0.141   0.840
E A  4P23C                 87    0.388  42.780  -0.608   0.018   0.047   0.935
E P  4P23C                 88    0.365  51.737  -1.245   0.004   0.085   0.910
E Q  4P23C                 89    0.412  73.530  -0.519   0.004   0.420   0.576
B A  4P23C                 90    0.092  10.160  -1.606   0.004   0.616   0.381
E T  4P23C                 91    0.269  37.324   0.305   0.002   0.816   0.182
B V  4P23C                 92    0.061   9.314  -1.107   0.001   0.900   0.099
B F  4P23C                 93    0.194  38.856  -0.035   0.002   0.816   0.182
B P  4P23C                 94    0.257  36.511  -1.562   0.022   0.552   0.426
E K  4P23C                 95    0.368  75.718  -0.629   0.020   0.205   0.775
E S  4P23C                 96    0.472  55.318  -1.116   0.018   0.088   0.893
E P  4P23C                 97    0.500  70.936  -1.555   0.018   0.088   0.893
E V  4P23C                 98    0.505  77.588  -2.183   0.053   0.043   0.903
E L  4P23C                 99    0.534  97.794  -1.857   0.018   0.047   0.935
E L  4P23C                100    0.387  70.860  -2.478   0.018   0.047   0.935
E G  4P23C                101    0.596  46.937  -2.343   0.018   0.047   0.935
E Q  4P23C                102    0.446  79.745  -1.457   0.018   0.088   0.893
E P  4P23C                103    0.484  68.736  -0.567   0.022   0.359   0.619
B N  4P23C                104    0.101  14.742  -0.869   0.087   0.683   0.230
B T  4P23C                105    0.182  25.299   0.451   0.085   0.775   0.140
B L  4P23C                106    0.041   7.562  -0.134   0.072   0.855   0.073
B I  4P23C                107    0.122  22.496   0.296   0.072   0.855   0.073
B C  4P23C                108    0.051   7.090  -0.395   0.056   0.913   0.030
B F  4P23C                109    0.118  23.663   0.666   0.056   0.913   0.030
B V  4P23C                110    0.022   3.443   0.179   0.085   0.775   0.140
B D  4P23C                111    0.228  32.898  -0.486   0.074   0.484   0.442
E N  4P23C                112    0.396  58.018  -0.925   0.021   0.279   0.699
B I  4P23C                113    0.154  28.527  -1.550   0.019   0.141   0.840
E F  4P23C                114    0.274  55.012   0.195   0.004   0.085   0.910
B P  4P23C                115    0.177  25.045  -1.352   0.004   0.138   0.858
E P  4P23C                116    0.364  51.623  -0.236   0.005   0.336   0.660
E V  4P23C                117    0.347  53.334  -0.002   0.003   0.718   0.279
B I  4P23C                118    0.030   5.550   0.077   0.001   0.900   0.099
B N  4P23C                119    0.240  35.165   0.801   0.001   0.959   0.040
B I  4P23C                120    0.026   4.884   0.785   0.000   0.983   0.017
B T  4P23C                121    0.169  23.399   1.797   0.000   0.983   0.017
B W  4P23C                122    0.052  12.458   0.866   0.001   0.959   0.040
B L  4P23C                123    0.237  43.303   1.139   0.000   0.983   0.017
B R  4P23C                124    0.184  42.090   0.305   0.001   0.900   0.099
E N  4P23C                125    0.551  80.608  -0.589   0.005   0.045   0.951
E S  4P23C                126    0.686  80.446  -1.170   0.005   0.015   0.979
E K  4P23C                127    0.531 109.165   0.482   0.004   0.616   0.381
E S  4P23C                128    0.639  74.844   0.331   0.003   0.718   0.279
B V  4P23C                129    0.195  30.018   0.710   0.021   0.451   0.528
E A  4P23C                130    0.473  52.081  -0.105   0.021   0.279   0.699
E D  4P23C                131    0.665  95.841  -0.290   0.019   0.141   0.840
E G  4P23C                132    0.682  53.666  -1.344   0.020   0.205   0.775
E V  4P23C                133    0.325  49.906  -0.238   0.021   0.451   0.528
E Y  4P23C                134    0.485 103.538  -0.547   0.023   0.655   0.322
E E  4P23C                135    0.379  66.229  -0.988   0.022   0.552   0.426
E T  4P23C                136    0.316  43.774  -2.185   0.022   0.359   0.619
E S  4P23C                137    0.560  65.632  -1.552   0.021   0.451   0.528
E F  4P23C                138    0.420  84.314  -0.793   0.022   0.552   0.426
E F  4P23C                139    0.340  68.318  -0.314   0.023   0.655   0.322
E V  4P23C                140    0.453  69.672  -1.096   0.004   0.514   0.481
E N  4P23C                141    0.472  69.115  -0.884   0.005   0.262   0.733
E R  4P23C                142    0.676 154.827  -1.922   0.005   0.045   0.951
E D  4P23C                143    0.661  95.265  -1.450   0.005   0.015   0.979
E Y  4P23C                144    0.327  69.859  -0.738   0.004   0.138   0.858
B S  4P23C                145    0.139  16.303   0.202   0.003   0.718   0.279
B F  4P23C                146    0.060  12.142   0.171   0.001   0.900   0.099
B H  4P23C                147    0.087  15.862   0.535   0.001   0.959   0.040
B K  4P23C                148    0.062  12.712  -0.090   0.001   0.959   0.040
B L  4P23C                149    0.114  20.837   0.369   0.001   0.959   0.040
B S  4P23C                150    0.037   4.336  -0.677   0.001   0.959   0.040
B Y  4P23C                151    0.139  29.726   0.532   0.001   0.959   0.040
B L  4P23C                152    0.037   6.848  -0.354   0.001   0.959   0.040
E T  4P23C                153    0.340  47.172   0.619   0.001   0.959   0.040
B F  4P23C                154    0.050   9.935   0.761   0.003   0.718   0.279
E I  4P23C                155    0.329  60.846   1.234   0.005   0.336   0.660
E P  4P23C                156    0.374  53.085  -0.132   0.004   0.085   0.910
E S  4P23C                157    0.690  80.845   0.416   0.005   0.015   0.979
E D  4P23C                158    0.646  93.074   0.457   0.005   0.015   0.979
E D  4P23C                159    0.667  96.115  -0.368   0.005   0.015   0.979
E D  4P23C                160    0.456  65.767   0.233   0.004   0.138   0.858
E I  4P23C                161    0.466  86.247   1.809   0.003   0.718   0.279
B Y  4P23C                162    0.061  12.950   0.814   0.001   0.900   0.099
B D  4P23C                163    0.245  35.362   1.364   0.001   0.959   0.040
B C  4P23C                164    0.016   2.317   1.246   0.001   0.959   0.040
B K  4P23C                165    0.229  47.146   1.735   0.001   0.959   0.040
B V  4P23C                166    0.031   4.765   0.518   0.001   0.959   0.040
E E  4P23C                167    0.356  62.158   0.818   0.001   0.959   0.040
B H  4P23C                168    0.083  15.134  -0.287   0.002   0.816   0.182
E W  4P23C                169    0.569 136.845   0.076   0.004   0.197   0.799
E G  4P23C                170    0.527  41.514  -1.010   0.004   0.085   0.910
B L  4P23C                171    0.209  38.323   0.140   0.004   0.085   0.910
E E  4P23C                172    0.691 120.700   0.242   0.018   0.047   0.935
E E  4P23C                173    0.533  93.133   0.561   0.018   0.047   0.935
E P  4P23C                174    0.467  66.253  -0.187   0.019   0.141   0.840
B V  4P23C                175    0.191  29.387   0.360   0.023   0.655   0.322
E L  4P23C                176    0.486  88.932   0.404   0.018   0.846   0.136
E K  4P23C                177    0.360  74.011   1.670   0.018   0.846   0.136
E H  4P23C                178    0.513  93.387   1.542   0.003   0.718   0.279
E W  4P23C                179    0.356  85.570  -1.131   0.003   0.003   0.994

Explain the output.  Go back.
```

---
